# Comparison of Modified Ovillanta, Modified Gravid *Aedes* Trap, Biogents Gravid *Aedes* Trap and Biogents Sentinel for Capturing *Aedes aegypti* in Tanzania

**DOI:** 10.3390/insects17090955

**Published:** 2026-09-13

**Authors:** Sinaraha Ally Seiph, Johnson K. Swai, Aidi G. Lugenge, Frank S. C. Tenywa, Olukayode G. Odufuwa, Joseph B. Muganga, Jason Moore, Esther G. Kimaro, Sarah J. Moore, Jane Johnson Machange

**Affiliations:** 1 School of Life Sciences and Bio-Engineering, The Nelson Mandela African Institution of Science and Technology (NM-AIST), Tengeru P.O. Box 447, Tanzania; 2 Vector Control Product Testing Unit, Environmental Health and Ecological Science Department, Ifakara Health Institute, Bagamoyo P.O. Box 74, Tanzaniaoodufuwa@ihi.or.tz (O.G.O.); jmoore@ihi.or.tz (J.M.);; 3 Vector Biology Unit, Department of Epidemiology and Public Health, Swiss Tropical and Public Health Institute, Kreuzstrasse 2, 4123 Allschwil, Switzerland; 4 Faculty of Science, University of Basel, Petersplatz 1, 4001 Basel, Switzerland

**Keywords:** *Aedes aegypti*, mosquito traps, vector control, Ovillanta, Biogents Sentinel, gravid *Aedes* trap, modified gravid *Aedes* trap, yeast, vector surveillance, Tanzania

## Abstract

*Aedes aegypti* surveillance is essential for guiding arbovirus control strategies. The BG-Sentinel (BGS) trap is a widely used reference trap for monitoring adult *Ae. aegypti*, but its cost and need for electricity limit its use in resource-constrained settings. This study evaluated locally made, low-cost oviposition-based traps relative to the BGS trap. The Ovillanta trap was modified with internal adhesive surfaces to capture adult mosquitoes. Six trap types were evaluated at six outdoor locations, including (1) Ovillanta with yeast (2) Ovillanta with only water, (3) BG-Gravid *Aedes* Trap (BG-GAT) with yeast, (4) one modified GAT (1MoGAT) with yeast, (5) four modified GATs (4MoGAT) with yeast, and (6) a standard BGS trap with BG-Lure. Traps were deployed for three consecutive 24 h sampling periods before rotation. This process was repeated four times resulting in a total of 72 experimental days. The primary outcome was total number of captured female *Ae. aegypti*. The yeast-baited modified Ovillanta captured more *Ae. aegypti* than the other oviposition-based traps (*n* = 468), with catches comparable to the BGS trap (*n* = 497; IRR = 1.03, 95% CI: 0.76–1.41; *p* = 0.859). Removing yeast from the Ovillanta reduced captures by 31% (*n* = 321; IRR = 0.69, 95% CI: 0.51–0.95; *p* = 0.023). The BG-GAT (*n* = 95), 4MoGAT (*n* = 35), and 1MoGAT (*n* = 31) captured significantly fewer *Ae. aegypti* than the yeast-baited modified Ovillanta (all *p* < 0.0001). Around 95% of captured mosquitoes were unfed across all trap types. These findings suggest that the yeast-baited modified Ovillanta may provide a low-cost alternative for adult female *Ae. aegypti* surveillance, but further evaluation across diverse ecological and operational settings is needed.

## Simple Summary

*Aedes* mosquito surveillance is used to guide integrated vector management for arbovirus disease control. Although the Biogents Sentinel (BGS) trap is considered the standard trap for monitoring *Aedes aegypti*, its relatively high cost and reliance on a battery powered fan limit large-scale use in resource-constrained settings. This study evaluated the performance of lower-cost, oviposition-based traps relative to the standard BGS trap. This included the Biogents Gravid *Aedes* Trap (BG-GAT), a locally produced modified Gravid *Aedes* Trap (MoGAT) applied at different densities, either one or four together, and a modified Ovillanta trap. Oviposition traps were yeast-baited and the Ovillanta was modified to capture adult mosquitoes by incorporating internal adhesive surfaces. The modified Ovillanta with yeast captured adult female *Ae. aegypti* numbers similar to the standard BGS trap and outperformed all other gravid traps. These findings suggest that the modified Ovillanta is a promising candidate oviposition trap for further evaluation.

## 1. Introduction

Arbovirus diseases remain a major global public health challenge. Dengue is one of the fastest expanding vector-borne diseases in tropical and subtropical regions [1,2,3], with reported global cases having increased sharply more than five-fold over the past fifty years [3,4]. This expansion is associated with the spread of competent vectors, particularly *Aedes aegypti* [5], driven by factors such as unplanned urbanization, climate change [1,2], increasing human mobility [6], and increasing intercontinental trade [7]. Genetic evidence suggests that competent *Ae. aegypti* populations were reintroduced into Africa from the Americas and Asia [8], and human mobility may contribute to wider circulation of virus genotypes [9].

In sub-Saharan Africa, dengue transmission increased by more than 400% between 1990 and 2019 [10]. The African continent is estimated to have a dengue burden similar to Latin America, although only a tiny proportion of cases are reported [11]. The number of cases in sub-Saharan Africa is likely to further increase due to massive urban population growth. Therefore, there is a need for effective vector surveillance and control strategies.

Integrated vector management is the primary approach for dengue prevention [1,2,7]. Vector surveillance is a necessary component of this approach [12,13] because it supports proactive *Aedes* control and targeted deployment of interventions [14]. Reliable mosquito sampling tools are essential for identifying local vector species, understanding their ecology, and monitoring the impact of control programs [15]. Despite the rapid spread of dengue in Africa, systematic vector surveillance remains limited [3,16]. Several studies have evaluated the performance of traps for sampling *Ae. aegypti* mosquitoes [17,18,19,20]. However, evidence on the local performance of *Aedes* surveillance traps in Tanzania remains scarce, despite the country experiencing recurrent dengue outbreaks where all four dengue virus serotypes co-circulate [21,22,23]. Identifying affordable and locally adaptable surveillance tools is therefore critical for strengthening vector monitoring.

Various *Aedes* mosquito sampling techniques have been developed [15,24,25,26]. Among the most widely used methods is the BG-Sentinel (BGS) [7,27]. It is a fan-powered device that uses chemical lures to attract host-seeking mosquitoes and is widely considered the gold-standard method for sampling *Aedes* populations [7,27]. However, the BGS trap is relatively expensive and requires electricity or batteries for operation, limiting its large-scale use in resource-constrained settings [19].

Other potentially more cost-effective trapping methods are lethal ovitraps (gravid traps), including the Gravid *Aedes* Trap (GAT) [25,28], Autocidal Gravid Ovitrap (AGO) [17], and Ovillanta trap [29]. Gravid traps target gravid mosquitoes, female mosquitoes that have already taken a blood meal and developed mature eggs (gravid) and are seeking suitable habitats to lay their eggs (oviposition) [30]. These traps operate as passive devices that exploit the attraction of mosquitoes to water and organic infusions that mimic breeding habitats [18,31]. Mosquitoes entering these traps are typically retained on adhesive surfaces or insecticide-treated materials inside the trap chamber [15]. The Ovillanta trap [29,32,33] is made of used vehicle tires, and is designed to attract mosquitoes to lay their eggs. The resulting eggs and larvae can then be collected and identified to species for surveillance, or the contents of the Ovillanta trap can be dumped or used with larvicide to reduce mosquito densities [34]. Such traps apply a “lure-and-kill” strategy that can reduce adult mosquito populations if they are deployed at high density, while also providing surveillance data [19,21]. *Aedes* mosquitoes also exhibit “skip oviposition”, a behavior in which females distribute a single batch of eggs across multiple larval habitats to enhance the probability of egg survival [35]. This behavior makes the mosquitoes more vulnerable to lethal ovitraps.

In this study, two locally constructed gravid traps were evaluated alongside the commercially available BGS [27] and BG-GAT [28,36]. The first was a modified Gravid *Aedes* Trap (MoGAT), with the internal chamber lined with insecticide-treated netting as a killing agent [24]. The second was a modified Ovillanta trap, which incorporated an adhesive surface mounted on a rectangular board and inserted on both sides of the upper and lower tires to retain adult mosquitoes entering to rest or lay eggs. In addition, the Ovillanta was tested with and without yeast to evaluate whether yeast enhanced trap catches.

This study aimed to evaluate the performance of low-cost, locally developed *Aedes* surveillance traps under field conditions in Bagamoyo, Tanzania and to identify more scalable and affordable surveillance tools for routine *Ae. aegypti* monitoring in resource-limited settings.

## 2. Materials and Methods

### 2.1. Study Area

Bagamoyo is a tropical coastal area located at 6°25′59.9988″ S latitude and 38°54′0.0072″ E longitude [37], approximately 65 km north of Dar es Salaam, the country’s economic hub. Bagamoyo experiences a bimodal climate with two rainy seasons, heavy rains from March to May and short rains from November to December, with occasional rainfall throughout the year, amounting to approximately 1200 to 2100 mm annually. Temperatures range between 22 °C and 33 °C, with an average relative humidity of 73% [38]. The area is characterized by unplanned settlements, poor sanitation and inadequate water storage, all of which create favorable conditions for larval development of *Aedes* mosquitoes. The study was conducted outdoors within the grounds of a hotel in Bagamoyo Town, selected for its accessibility and the abundance of *Aedes* larval habitats. The hotel was chosen as the study site because it provided a controlled environment with conditions conducive to the survival and proliferation of *Ae. aegypti*.

### 2.2. Experimental Design and Procedure

**Figure 1:**
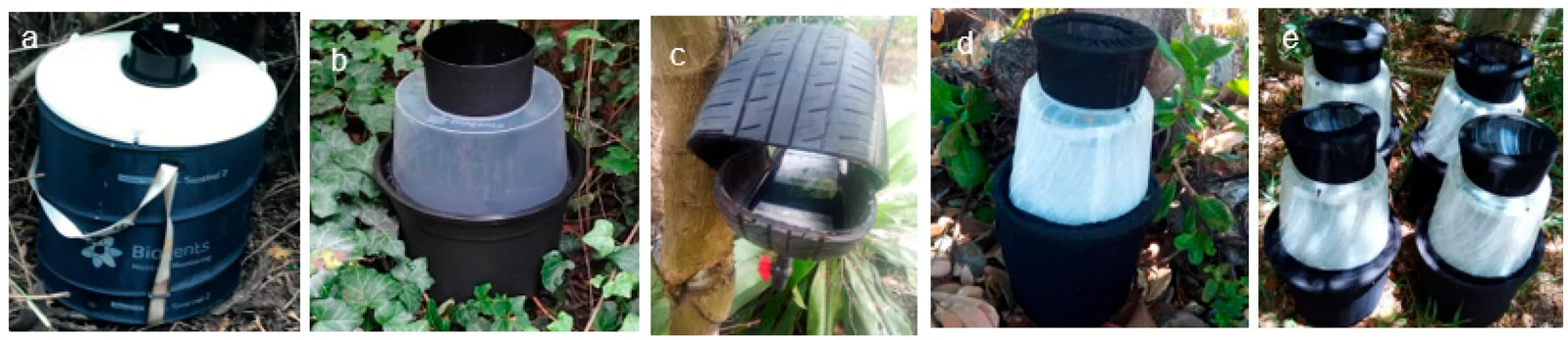
*Aedes* monitoring tools: (**a**) Biogents Sentinel (BGS) trap (standard trap), (**b**) Biogent Gravid *Aedes* Trap (BG-GAT), (**c**) modified Ovillanta trap, (**d**) single-unit modified *Aedes* Gravid Trap (1MoGAT), and (**e**) four-unit modified Gravid *Aedes* Trap (4MoGAT).

An experimental field study was conducted between October and December 2025 over 72 days. Traps were deployed at six fixed outdoor locations within the hotel grounds, approximately 15 m apart and positioned in shaded areas [24]. Six trapping methods were evaluated: (1) BGS with BG-Lure, (2) BG-GAT with yeast, (3) modified Ovillanta trap with yeast, (4) modified Ovillanta trap with water, (5) single modified Gravid *Aedes* Trap (1MoGAT) with yeast, and (6) four-unit modified GAT (4MoGAT) with yeast (Figure 1). Each trap configuration was rotated through all six locations to minimize confounding by site and sampling day. At each of the six locations, the traps were deployed for three consecutive 24 h sampling periods before being rotated to a new location (6 locations × 3 days = 18 days for one experimental round). The rotation was repeated four times throughout the study, resulting in a total of 72 experimental days (18 days × 4 rounds). All traps operated continuously for 24 h intervals and were serviced daily at 10:00 h [24]. Captured mosquitoes were collected into paper cups, labeled by trap, location and date, and transported to the Ifakara Health Institute (IHI) laboratory at the Kingani facility in Bagamoyo for morphological identification [39].

### 2.3. Traps and Attractant Development

#### 2.3.1. Biogents Sentinel (BGS) Trap

The BGS trap (Biogent GmbH, Regensburg, Germany) is shown in Figure 1a. As described by Krockel et al., 2006 [36], the BGS trap targets host-seeking mosquitoes through a funnel-shaped intake connected to a 12 V fan that generates downward airflow, pulling mosquitoes into a collection bag [6]. During field deployment, the trap was baited with BG-lure (Biogent AG, Regensburg, Germany), a synthetic blend of lactic acid, caproic acid, and ammonia mimicking human odor [20,36]. The lure was placed inside the BGS trap and used throughout the 72-day experimental period, remaining within the manufacturer’s recommendation [27].

#### 2.3.2. Biogents Gravid *Aedes* Trap (BG-GAT)

The BG-GAT [40] was used as a commercially available gravid trap comparator (Figure 1b). The BG-GAT comprises five main components: (1) a matte-finished black bucket base that contains the lure; (2) a translucent chamber formed by an inverted translucent plastic container fitted securely into the base; (3) a 1 mm black nylon mesh barrier, positioned between the chamber and the base; (4) a black funnel entry point positioned at the top of the translucent chamber; and (5) a BG-GAT sticky card. The sticky card was replaced daily, and captured mosquitoes were recovered from the removed sticky cards for identification.

#### 2.3.3. Modified Ovillanta

The Ovillanta previously described by Ulibarri et al. (2017), and Umar et al. (2020) [29,41] consists of two 50 cm sections of discarded vehicle tires arranged in a “mouth-like” configuration as shown in Figure 1c. The unit consists of two tire sections: a base for the attractant solution, fitted with a drainage tap to facilitate the weekly replacement of the lure, and an upper section protecting the interior from rain and debris.

For this study, the modified Ovillanta trap was adapted by incorporating an industrial adhesive surface (FICS Flyroll) to capture adult mosquitoes. Two rectangular fiberboard frames were first constructed to fit in both sides of the Ovillanta base. The adhesive sheets were cut with a 1 mm overhang to eliminate non-adhesive landing surfaces, mounted onto each fiberboard with the sticky side facing outward, and secured with tape. Each completed unit was handled as a single piece and inserted into both openings of the trap base. Adult mosquitoes entering for resting or oviposition were captured upon contact with adhesive surface. After 24 h, the frames were collected, and then the captured mosquitoes were removed from adhesive surface using forceps, placed in labeled paper cups, and transported to the laboratory.

**Figure 2:**
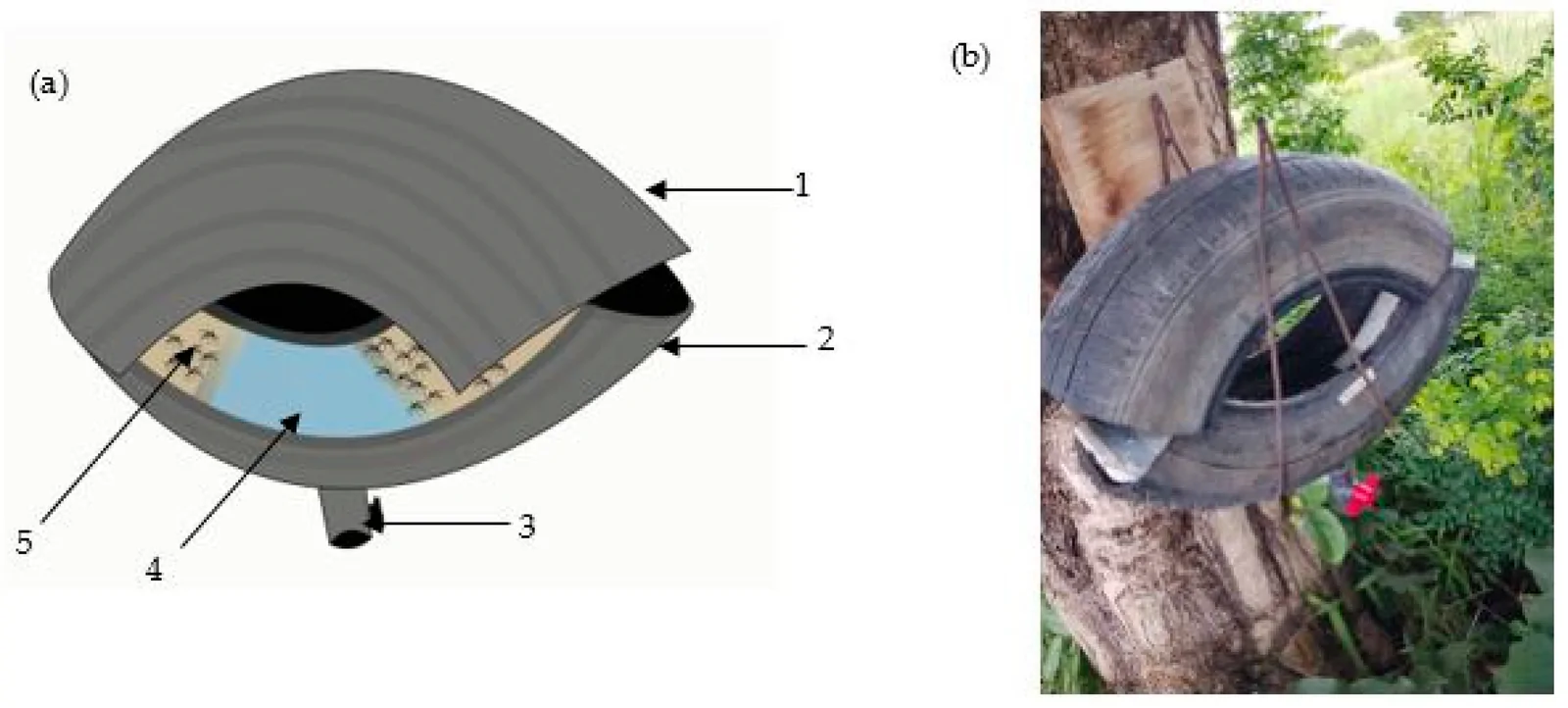
Modified Ovillanta trap: (**a**) schematic diagram for a modified Ovillanta; (1) 50 cm piece of tire used as trap cover; (2) 50 cm piece of tire used as trap base; (3) the water tap connected to the drainage pipe; (4) the disclosed trap infusion (yeast or water); and (5) the adhesive surface mounted on a fiberboard in both side of trap and (**b**) modified Ovillanta in the field setting.

Because no larvicide was used, any larvae that emerged were controlled by filtering the infusion solution weekly through a clean white lightweight cotton cloth. After filtration, the solution was returned to the trap. During the first sampling round (18 days), all collected larvae were reared to adulthood for species identification, and all emerging adults were identified as *Ae. aegypti*. In subsequent sampling rounds, larvae collected during filtration were buried in the ground (Figure 2b) [29].

#### 2.3.4. Modified Gravid *Aedes* Trap (MoGAT)

**Figure 3:**
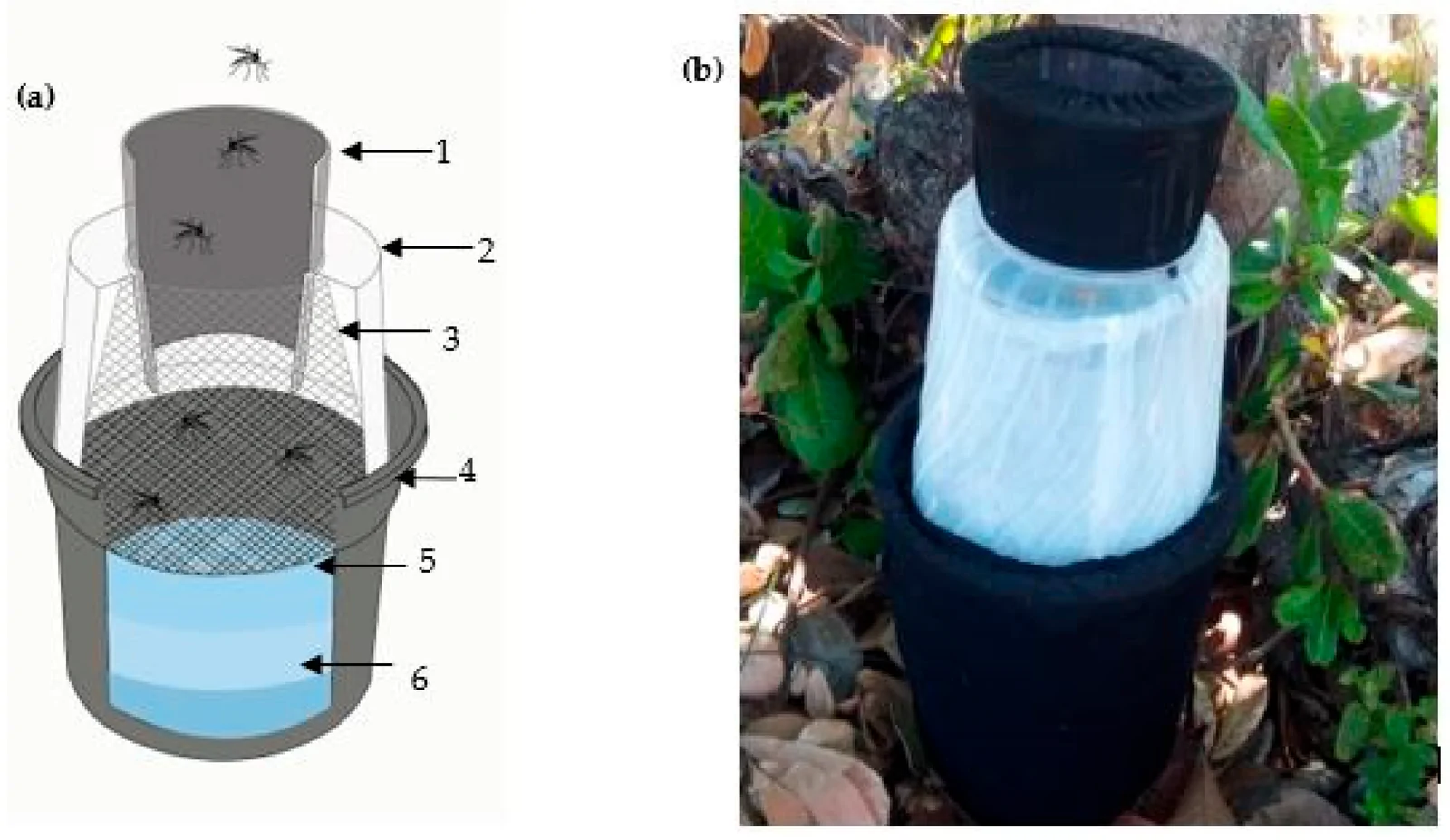
Modified Gravid *Aedes* Trap (MoGAT). (**a**) Modified GAT schematic diagram: (1) bucket of 3 L; (2) translucent 10 L bucket; (3) insecticide-treated net; (4) 16 L bucket; (5) 1 mm screen mesh; and (6) trap infusion. (**b**) Modified GAT in the field setting.

The MoGAT was constructed as previously described by Machange et al. 2024 [24] (Figure 1d). The trap consists of six components, (1) a three-liter (L) bucket with its base removed, covered in black cloth, and used as the mosquito entry point, (2) an inverted translucent ten-liter (L) bucket lined with (3) bifenthrin-treated net manufactured by VKA Polymers Ltd., (4) a sixteen-liter (L) bucket serving as the base, covered with black cloth and containing the lure, with drainage holes positioned above the three-liter level to prevent overfilling, (5) a layer of black mosquito mesh (1 mm) placed between the translucent bucket and the base to block mosquito access to the infusion, and (6) a trap infusion (Figure 3).

For this study, one modified GAT (1MoGAT) referred to the deployment of a single-unit modified GAT at a sampling location, whereas 4MoGAT referred to the deployment of four identical modified GATs positioned together at one location, with mosquitoes captured by all four traps combined and treated as a single experimental unit (Figure 1e). Both configurations were evaluated to determine whether increasing the number of modified GATs at a sampling point improved mosquito trap capture compared with a single trap deployment.

#### 2.3.5. Lure Preparation

To increase the attractiveness of gravid traps (BG-GAT, 1MoGAT, 4MoGAT, and Ovillanta) for mosquitoes, an infusion producing fermentation-derived volatiles was prepared by dissolving 22 g of dry baker’s yeast in 12 L of tap water. The solution was prepared as described by Machange et al. (2024); briefly, it was placed in a sealed container and away from direct sunlight for three days with manual agitation performed once daily with no additional substrate [24,42]. For field deployment, 2 L of the fermented attractant was added to each oviposition (Ovillanta and BG-GAT), and 3 L to each MoGAT trap, while control Ovillanta traps contained 2 L of water only; the lure was replaced every 14 days. The volume used and replacement interval followed Ulibarri et al. 2016 [29], and Machange et al. 2024 [24]; different replacement intervals were not experimentally compared in this study.

### 2.4. Data Management and Statistical Analysis

All data were collected into paper forms, double-entered into Microsoft Excel spreadsheet version 16, and cross-checked for duplicate entries and discrepancies before being imported into STATA/MP 18.0 (Stata Statistical Software: Release 18. College Station, TX, USA: Statacorp, LLC.) for analysis [43]. Any duplicate or inconsistent records were verified against the original field forms and corrected. Descriptive statistics were performed to estimate the geometric mean with 95% confidence intervals (CI) of female *Ae. aegypti* species for each trap in the field.

Mixed-effect negative binomial regressions were performed to compare the number of mosquitoes captured between the traps. Due to the low numbers of gravid and blood-fed individuals, analyzing separate physiological stages was unfeasible; therefore, the model focused on total female mosquitoes. Trap type and sampling points were included in the model as fixed effects while experimental day was included as a random effect to account for daily heterogeneity in mosquito densities.

Bland–Altman plots were used to evaluate agreement in female *Ae. aegypti* catches between the BGS and the modified Ovillanta with yeast infusion, as these were the two highest performing traps for capturing adult female *Ae. aegypti*. The mean difference and 95% limits of agreement were calculated for each comparison. Density-dependent effects were assessed by examining the relationship between the differences in paired trap catches and their corresponding mean catches, with significant trends indicating density-dependent bias.

## 3. Results

A total of 5051 mosquitoes were collected over 72 sampling nights; of these, 61.7% (*n* = 3115) were *Culex quinquefasciatus* and 38.2% (*n* = 1936) were *Ae. aegypti*. The majority of *Cx. quinquefasciatus* were captured by the BGS, which accounted for 88% of the female *Culex* collected, whereas the other trap types contributed substantially fewer individuals, (Figure S1). Of the *Ae. aegypti* captured, 74.6% (1447/1936) were female and 95% of the females (1368/1447) were unfed. The physiological status of the captured mosquitoes is summarized in Table S1. Subsequent analyses were restricted to total female *Ae. aegypti* only.

The BGS captured the highest number of female *Ae. aegypti*, representing 34.4% of total catch, followed by yeast-baited Ovillanta 32.3% and water-only Ovillanta 22.2%. The remaining oviposition traps captured substantially lower proportions of mosquitoes (BG-GAT 6.6%, 1MoGAT 2.1% and 4MoGAT 2.4%, (Table 1)).

The yeast-baited Ovillanta captured a similar number of female *Ae. aegypti* compared to BGS traps (IRR = 1.03, [95%CI: 0.76–1.40], *p* = 0.859), while capturing significantly more mosquitoes than the other gravid traps (all *p* < 0.0001) (Table 1). Without yeast infusion, Ovillanta catches decreased by 31% (IRR =0.69, [95% CI: 0.51–0.95], *p* = 0.023).

The commercial BGS captured significantly more mosquitoes than Ovillanta with water (IRR = 0.67, [95% CI: 0.49, 0.92], *p* = 0.013), and differences were not significant compared with Ovillanta with yeast (IRR = 0.97, [95%CI: 0.71, 1.32], *p* = 0.859 (Table 2)).

The commercial BG-GAT captured more mosquitoes (*n* = 95) than the 1MoGAT (*n* = 31, IRR = 0.37, [95%CI: 0.22–0.60], *p* < 0.0001) and 4MoGAT (*n* = 35, IRR = 0.32, [95%CI: 0.20–0.53], *p* < 0.0001), (Table 3).

**Figure 4:**
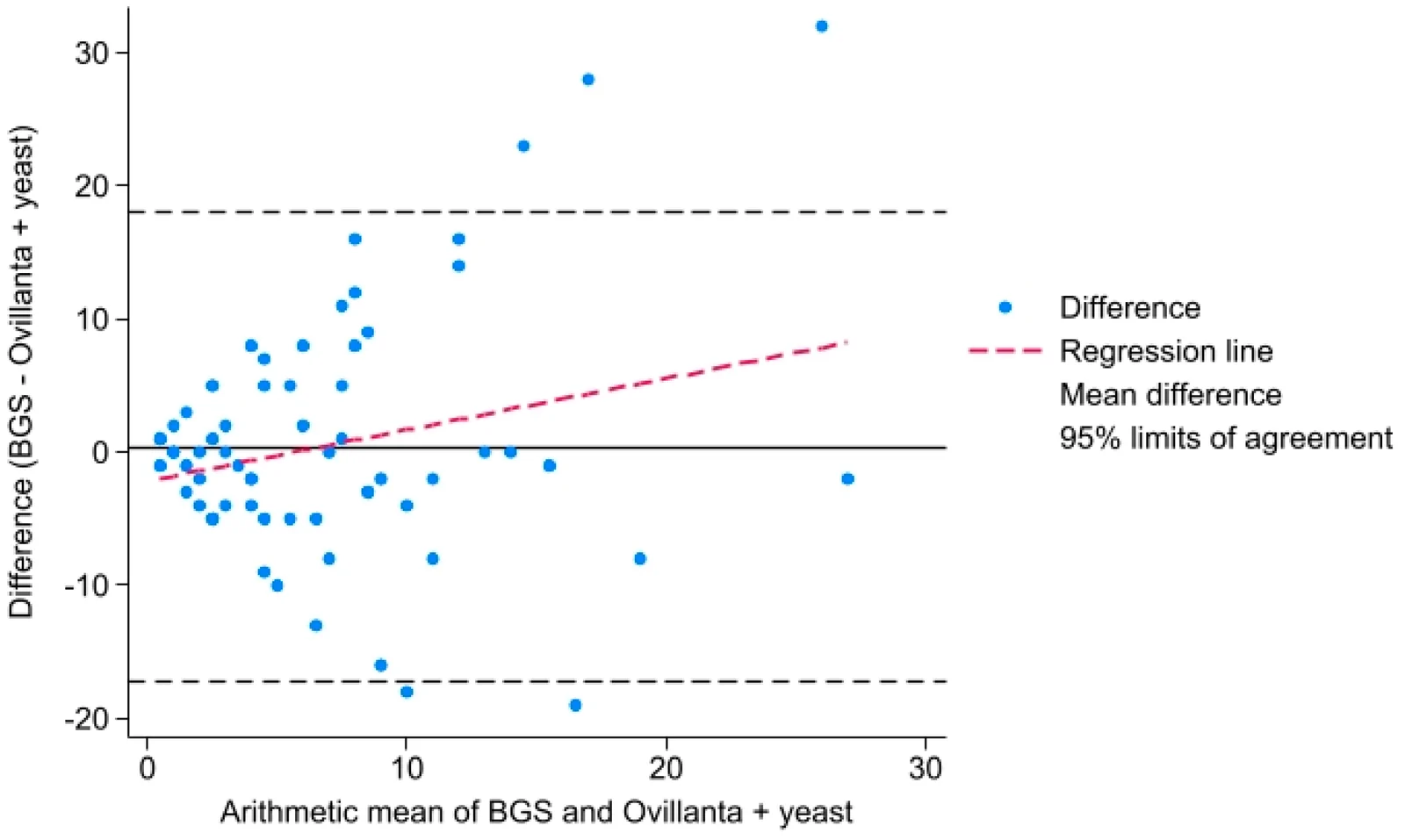
Bland−Altman plot assessing agreement and density-dependent bias between the Biogents Sentinel trap and Ovillanta with yeast infusion for capturing adult female *Aedes aegypti* mosquitoes under field conditions. The solid horizontal line represents the mean difference (bias), dashed lines indicate the 95% limits of agreement, and the fitted regression line indicates the density-dependent relationship between the two trapping methods.

The Bland–Altman analysis showed limited agreement between BGS and Ovillanta with yeast infusion, with a mean difference of 0.40 mosquitoes, but the limits of agreement were wide, from −17.29 to 17.29, indicating substantial daily variability in mosquito capture between the trap types (Figure 4). The positive regression slope indicated density-dependent bias. These findings indicate that although the yeast-baited modified Ovillanta and BGS traps produced similar total catches, they may not be directly interchangeable for estimating daily mosquito abundance, and further evaluation is warranted.

## 4. Discussion

Effective surveillance of *Aedes aegypti* is essential for guiding dengue prevention and control efforts, particularly in resource-limited settings where sustainable monitoring tools are needed [44]. In this study, the trapping performance of the modified Ovillanta, with and without yeast, was compared with several trapping methods, including 1MoGAT, 4MoGAT, BG-GAT, and the widely used reference trap (BGS trap), using a Latin square design that controlled for spatial and temporal variation [45]. The yeast-baited modified Ovillanta captured a similar total number of female *Ae. aegypti* to the BGS trap and substantially more than the BG-GAT and modified GAT designs. However, wide limits of agreement between the BGS and modified Ovillanta indicate that similar total catches do not necessarily imply that the traps are interchangeable for estimating daily vector density. The variation may reflect differences in trapping mechanisms, behavioral targets, and attractants. Therefore, the BGS and Ovillanta comparison represents a combined trap–lure effect rather than trap design alone.

The Ovillanta was originally developed as an oviposition device to collect *Aedes* eggs and thereby reduce subsequent mosquito generations [29,32,33]. Previous studies have shown that this trap effectively attracts gravid *Ae. aegypti* mosquitoes; however, because it was primarily designed to collect eggs, it provides a limited capacity for monitoring adult mosquito populations and detecting arboviruses [41]. In this study, the Ovillanta was modified to capture adult mosquitoes by incorporating internal adhesive surfaces [19,28], enabling direct capture of adult *Ae. aegypti*. The commercial BG-GAT similarly used a BG-GAT adhesive surface, while the MoGAT was equipped with a bifenthrin-treated net for adult mosquito retention.

### 4.1. The Influence of Trap Design of Mosquito Capture

The superior performance of modified Ovillanta trap is likely explained by the combined influence of visual and olfactory cues. Because the water-filled Ovillanta captured a significantly high number of mosquitoes compared to other ovitraps baited with yeast (BG-GAT and modified GATs), its relatively higher mosquito capture is partially attributed to the trap design [33]. Tires are preferred habitats for *Ae. aegypti* [46], due to water retention, shade, organic matter, and visual cues (black color) [19]. In addition, the adhesive sticky cards installed inside the trap effectively retain mosquitoes upon contact, thereby reducing escape probability [19,28,47].

When compared with unbaited (water-filled) Ovillanta, yeast-baited Ovillanta captured a substantially higher number of *Ae. aegypti*. Water acts as a lure by providing oviposition signals for gravid females [48]. However, the addition of yeast significantly enhances oviposition attraction in *Aedes* mosquitoes by acting as a nutrient-associated cue [42], leading to significantly higher trap attractiveness than water alone [49]. These differences in attraction mechanisms and mosquito behavior may also explain the wide limits of agreement observed when comparing the modified Ovillanta with a host-seeking trap (BGS).

Lower mosquito captures in the BG-GAT compared with the BGS trap are consistent with previous studies [1,7,17,20,24,28]. The BGS employs a fan-driven airflow to actively draw mosquitoes into a collection bag [27], whereas the BG-GAT relies on passive entry without mechanical suction. The BGS traps can attract and draw in mosquitoes from distances of several meters under field conditions [50]. Its performance is likely attributed to the combination of synthetic human-odor lures, comprising a blend of lactic acid, caproic acid, and ammonia [27]. These volatile compounds create active plumes that, combined with the trap suction, actively draw mosquitoes into the trap [31,42]. This pattern is consistent with reports from West Africa [51], Cameroon [52], and Ghana [53,54].

Comparative studies indicate that the performance of different trap types varies across ecological contexts [22], likely reflecting variations in mosquito ecology [55]. For instance, in Brazil, GATs captured fewer adult mosquitoes overall but a higher proportion of gravid individuals compared to BGS traps [17], whereas in Guinea, gravid traps collected similar numbers of gravid mosquitoes but fewer unfed *Aedes* than BGS traps [56]. These findings underscore the importance of evaluating trap performance within specific contexts, taking into account ecological suitability in addition to operational feasibility and cost-effectiveness.

### 4.2. Operational Limitations of the Modified Ovillanta

Although the Ovillanta trap caught more *Aedes* than other gravid traps, environmental factors such as heavy rainfall combined with high winds occasionally reduced trap capture by moisture-logging the adhesive surfaces. This was similarly observed in the BG-GAT, highlighting the need for weather-resistant adhesive materials in future field optimizations.

Larvae were occasionally detected in the infusion in Ovillanta traps. Upon rearing them to adulthood, all adult mosquitoes (*n* = 763) were confirmed as *Ae. aegypti* mosquitoes, indicating that some gravid females successfully oviposited without contacting the internal adhesive surfaces. This suggests that if traps are left unattended, they may become potential larval habitats for *Ae. aegypti.* The potential use of long-lasting larvicides, such as spinosad, Bti, or pyriproxyfen, could be explored in future studies to prevent potential larval development when traps are left unattended [34,57], thereby strengthening the trap’s utility for both integrated surveillance and vector control [57].

### 4.3. Performance of BG-GAT and Other GAT Variants

The superior performance of BG-GAT observed compared to modified GAT may be attributed to its specific trap design. The BG-GAT uses an adhesive sticky board, which retains mosquitoes immediately upon contact. The modified GAT used bifenthrin-treated net as a killing agent [24], which requires sufficient contact time for a lethal exposure. Further work to explore low-cost adhesives or other alternatives to optimize the locally produced GAT is warranted.

Since *Aedes* are skip ovipositors [58], it was hypothesized that increasing the number of traps may increase the radius of attraction and increase the capture rate [19]. Interestingly, increasing the number of modified GATs from one to four did not yield higher capture rates; instead, this study found that the mosquitoes were observed resting in the sheltered gaps between the traps. A similar study conducted in this setting compared BGS and 4MoGAT, stationed at different locations (1MoGAT per location), and suggested that more replicates of GAT at multiple locations and pooling data [24] is a more effective approach than stationing four traps at one location, agreeing with findings from Puerto Rico [19].

### 4.4. Study Limitations

This study had several limitations. Firstly, it was conducted at a single commercial site in Bagamoyo over one sampling period, and the findings may not be generalizable to other ecological, seasonal, or community settings. Therefore, multi-site and multi-season studies are needed to determine whether the observed differences in trap performance remain consistent under different environmental conditions. Secondly, gravid traps have the advantage of capturing females that have already taken a blood meal and are therefore more likely to carry arboviruses, such as dengue [28,40]. Consequently, these traps can also contribute to arbovirus surveillance through xenomonitoring, which is the screening of field-collected vectors for pathogens to provide indirect evidence of pathogen circulation in the host population [12,13]. Very few blood-fed and gravid *Ae. aegypti* were captured and virus detection was not conducted. Therefore, evaluating whether these traps are suitable for xenomonitoring requires testing of all captured female *Ae. aegypti*, regardless of their blood meal status, and warrants further investigation. Thirdly, the rainfall and wind occasionally affected adhesive surfaces, indicating that trap performance may vary under different weather conditions and that design modifications involving more weather-resistant materials may be needed. In addition, the presence of predators (ants) within BG-GATs, likely entering through the overflow outlet, may have contributed to mosquito loss. These observations highlight the importance of appropriate trap allocation and placement to minimize ants’ interference and other environmental factors that may influence mosquito retention and trap performance. Fourthly, larvae were detected in some Ovillanta traps, showing that traps could become productive larval habitats if not inspected and serviced regularly. The study was an early phase efficacy study and more data are needed in additional settings to confirm the findings before evaluating community acceptability, operational feasibility at scale, cost-effectiveness and dengue virus detection in captured mosquitoes.

Although this study focused on *Ae. aegypti*, *Culex quinquefasciatus* accounted for a substantial proportion of mosquitoes collected. The predominance of female *Culex* in the BGS compared with the other trap types suggests that the BGS is a potential tool for the surveillance of *Culex* and other medically important mosquito species.

Based on the findings of this study, and other studies all showing positive results [29,32,33], Ovillanta provides a practical and efficient tool for monitoring dengue vectors, offering key operational benefits such as durability, low cost (<10 $USD—designed from locally available recycled materials) and no reliance on electricity [29]. Taken together, these findings support further evaluation of the modified Ovillanta as a low-cost, electricity-free tool for adult female *Ae. aegypti* surveillance.

## 5. Conclusions

The yeast-baited modified Ovillanta captured a similar number of female *Ae. aegypti* as the BGS trap and substantially higher numbers than the other oviposition-based traps. Yeast substantially improved the Ovillanta’s performance. The commercial BG-GAT outperformed locally modified GATs, and increasing the number of Mo-GAT from one to four in a single location did not improve catches. These findings support further evaluation of the yeast-baited modified Ovillanta across additional ecological, seasonal and operational settings to determine whether it can provide a practical, low-cost tool for routine adult female *Ae. aegypti* surveillance.

## Figures and Tables

**Table 1:** Geometric mean catches and incidence rate ratios (IRRs) of female *Aedes aegypti* captured by yeast-baited modified Ovillanta relative to other trap types in Bagamoyo, Tanzania, during the 72-night study period (October–December 2025).

Trap	*N*	*n*	Mean (95%CI)	IRR (95%CI)	*p*-Value
Ovillanta + yeast	72	468	5.13 (4.11, 6.40)	1.00	
Ovillanta + water	72	321	3.86 (3.17, 4.70)	0.69 (0.51, 0.95)	0.023
BG-GAT + yeast	72	95	1.92 (1.52, 2.43)	0.19 (0.13, 0.27)	<0.0001
1MoGAT + yeast	72	31	1.25 (0.99, 1.57)	0.06 (0.04, 0.10)	<0.0001
4MoGAT + yeast	72	35	1.41 (1.11, 1.81)	0.07 (0.04, 0.11)	<0.0001
BGS + BG-lure	72	497	4.81 (3.68, 6.28)	1.03 (0.76, 1.40)	0.859

*N* = total number of days; *n* = total number of mosquitoes captured; Mean = geometric mean; IRR is incidence rate ratio.

**Table 2:** Geometric mean catches and incidence rate ratio (IRR) of female *Aedes aegypti* mosquitoes captured by Biogents Sentinel (BGS) trap relative to other trap types in Bagamoyo, Tanzania, during the 72-night study period (October–December 2025).

Traps	*N*	*n*	Mean (95%CI)	IRR (95%CI)	*p*-Value
BGS + BG-lure	72	497	4.81 (3.68, 6.28)	1.00	
Ovillanta + yeast	72	468	5.13 (4.11, 6.40)	0.97 (0.71, 1.32)	0.859
Ovillanta + water	72	321	3.86 (3.17, 4.70)	0.67 (0.49, 0.92)	0.013
4MoGAT + yeast	72	35	1.41 (1.10, 1.81)	0.07 (0.04, 0.10)	<0.0001
1MoGAT + yeast	72	31	1.24 (0.99, 1.57)	0.06 (0.04, 0.09)	<0.0001
BG-GAT + yeast	72	95	1.92 (1.52, 2.43)	0.18 (0.13, 0.26)	<0.0001

*N* = total number of days; *n* = total number of mosquitoes captured; Mean = geometric mean; IRR is incidence rate ratio.

**Table 3:** Geometric mean catches and incidence rate ratio (IRR) of female *Aedes aegypti* mosquitoes captured by Biogents Gravid *Aedes* Trap (BG_GAT) with yeast relative to other trap types in Bagamoyo, Tanzania, during the 72-night study period (October–December 2025).

Traps	*N*	*n*	Mean (95%CI)	IRR (95%CI)	*p*-Value
BG-GAT	72	95	1.92 (1.52, 2.43)	1.00	
Ovillanta + yeast	72	468	5.13 (4.11, 6.40)	5.31 (3.70, 7.63)	<0.0001
Ovillanta + Water	72	321	3.86 (3.17, 4.70)	3.70 (2.56, 5.35)	<0.0001
4MoGAT + yeast	72	35	1.41 (1.11,1.81)	0.37 (0.22, 0.60)	<0.0001
1MoGAT + yeast	72	31	1.25 (0.99, 1.57)	0.32 (0.20, 0.53)	<0.0001
BGS + BG-lure	72	497	4.81 (3.68, 6.28)	5.50 (3.82, 7.91)	<0.0001

*N* = total number of days; *n* = total number of mosquitoes captured; Mean = geometric mean; IRR is incidence rate ratio.

## Data Availability

All data generated or analyzed during this study are included in this published article and will be available upon request.

## Supplementary Materials

The following supporting information can be downloaded at https://doi.org/10.5281/zenodo.22179236, Table S1: Geometric means and incidence rate ratios (IRRs) of physiological stages of female *Aedes aegypti* mosquitoes captured at multiple sampling points within a hotel in Bagamoyo, Tanzania, during the 72-night study period (October–December 2025), Figure S1: Number of female *Culex* mosquitoes captured at multiple sampling points within a hotel in Bagamoyo, Tanzania, during the 72-night study period (October–December 2025).

## Abbreviations

The following abbreviations are used in this manuscript:

BG-GAT	Biogent Gravid *Aedes* Trap
BGS	Biogents Sentinel Trap
CHIKV	Chikungunya Virus
DENV	Dengue Virus
ITN	Insecticide-Treated Nets
Mo-GAT	Locally Modified Gravid *Aedes* Trap
Mo-Ovillanta	Locally Modified Ovillanta
WHO	World Health Organization
1MoGAT	Single-Unit Modified Gravid *Aedes* Trap
4MoGAT	Four-Unit Modified Gravid *Aedes* Trap
